# Manipulating plasma thyroid hormone levels alters development of endothermy and ventilation in nestling red-winged blackbirds

**DOI:** 10.3389/fphys.2022.1027257

**Published:** 2022-11-29

**Authors:** Tushar S. Sirsat, Sarah K. G. Sirsat, Edwan R. Price, Megan Pineda, Edward M. Dzialowski

**Affiliations:** ^1^ Department of Physician Assistant Studies, Clarkson University, Potsdam, NY, United States; ^2^ Developmental Integrative Biology, Department of Biological Sciences, University of North Texas, Denton, TX, United States; ^3^ Department of Biology, SUNY Potsdam, Potsdam, NY, United States; ^4^ Green Godwit Consulting, Cleveland, OH, United States; ^5^ FUJIFILM Diosynth Biotechnologies Texas, College Station, TX, United States

**Keywords:** endothermy, thyroid hormone, ventilation, metabolic rate, altricial bird, Agelaius phoeniceus

## Abstract

Thyroid hormones are key regulators of development and metabolism in vertebrates. During the nestling period, young of altricial species transition from an ectothermic phenotype to an endothermic phenotype. Red-winged blackbirds are an altricial species that exhibit an increase in plasma 3,3’, 5-triiodo-L-thyronine (T_3_) levels during the first 5 days post-hatch (dph), begin to develop endothermic metabolic responses by 7 dph, and fledge within 10 days of hatching. We propose that thyroid hormones play an important role in regulating development of endothermy during the nestling period in altricial birds. To better understand the effects of thyroid hormones on endothermic metabolic development in an altricial species, we treated nestling red-winged blackbirds on 2, 3, and 5 dph with either methimazole (MMI) to induce hypothyroidism or supplemental T_3_ to induce hyperthyroidism. We then measured on 5, 7, and 9 dph morphology and whole animal O_2_ consumption ( ${\dot{V}o}_{2}$ ) and ventilation in the thermal neutral zone and during gradual cooling. Treatment of nestlings with MMI resulted in lower plasma T_3_ levels on 5 dph that recovered by 7 dph, while supplementing with T_3_ did not affect plasma T_3_ levels on 5, 7 and 9 dph. Treatment with MMI resulted in smaller nestlings with smaller hearts and structural characters such as wing chord and femur length, but larger lungs and kidneys. Treatment with T_3_ produced smaller nestlings with smaller body masses and shorter femur and tarsus lengths. The development of ${\dot{V}o}_{2}$ and ventilation endothermic responses to gradual cooling in MMI treated nestlings were delayed when compared with control nestlings. In 9 dph nestlings, hypothyroidism resulted in alterations in the responses of ventilation frequency and tidal volume to cooling when compared with the control nestlings. Supplemental T_3_ had no effect on the development of ${\dot{V}o}_{2}$ and ventilation in the thermal neutral zone or in response to cooling. Our data suggest plasma thyroid hormone levels play an active role in the systemic development of endothermic capacity and the development of ventilatory control. In the nestling avian, multiple systems develop in concert to produce an endothermic phenotype, but reduced thyroid hormone delays maturation of endothermic capacity.

## Introduction

Red-winged blackbirds (*Agelaius phoenicius*) are altricial birds that hatch with an ectothermic-like phenotype, naked without insulation and poor motor coordination, leaving them unable to effectively thermoregulate early in nestling development. During the first days of nestling life, energy is devoted towards growth, and hatchlings increase in body mass by 9–10 times their initial body mass, resulting in one of the fastest measured avian nestling growth rates (Olson, 1992). During these first days, nestlings allocate most of their energy towards growth and structural development and depend on their parents for thermoregulation (Olson, 1992; Olson et al., 1999). Red-winged blackbird nestlings gain greater endothermic capacity—observed as a metabolic thermogenic response to cold challenges—between 5 and 9 days post hatching (Olson, 1992; Sirsat et al., 2016). During this transition from incipient to fully mature endothermy, various systems such as whole-body metabolism, body temperature regulation, pulmonary ventilation, organ masses, and skeletal mitochondrial function undergo drastic changes (Sirsat et al., 2016; Price and Dzialowski, 2018).

Thyroid hormones play a prominent role in regulating metabolism and energy expenditure of birds (Decuypere et al., 2005) and exert effects on key physiological functions involved in energy use of tissues (Yen, 2001), including development of body systems and obligatory metabolic heat production of endothermic species (Breall et al., 1984; McNabb, 2007; Little and Seebacher, 2014). During cold exposure, endotherms increase their metabolic rates and heat production to thermoregulate within a given range of body temperatures. The capacity for adult metabolic responses are mediated in part by thyroid hormones action on skeletal muscle and brown adipose tissue (Iwen et al., 2018).

Thyroid hormones are important for a number of developmental processes in birds during hatching and nestling development (De Groef et al., 2013). A number of recent studies examined the influence of maternal thyroid hormones passed on in the egg on subsequent development in altricial species development (Hsu et al., 2020; Sarraude et al., 2020; Cossin-Sevrin et al., 2022). Elevating egg yolk thyroid hormones resulted in earlier hatching and larger hatchlings in collared flycatchers (*Ficedula albicollis*) (Hsu et al., 2019) but had no effect in the great tit hatchling (*Parus major*) (Cossin-Sevrin et al., 2022). The effect on the collared flycatcher disappeared quickly in the developing nestling. Alternatively, inhibition of thyroid hormones during the nestling period of the zebra finch (*Taeniopygia guttata*) resulted in decreased growth and altered behavior (Rainwater et al., 2008). Based on these studies, we predict that thyroid hormones produced by the nestling are more important regulators of metabolic development than those supplied in the yolk by the mother.

Thyroid levels and hypothalamic-pituitary-thyroid (HPT) axis control in altricial species like the red-winged blackbird increase in parallel with development of endothermic capacity (Olson et al., 1999). Red-winged blackbird nestling T_3_ plasma levels peak at 4 days post-hatch (dph) and remain elevated through day 8 of post hatching life. This developmental trajectory for plasma T_3_ levels is consistent with documented rises in plasma T_3_ in other altricial species such as ring-necked doves, great tits, and European starlings (McNabb and Cheng, 1985; Schew et al., 1996; Silverin and Rudas, 1996; Výboh et al., 1996). Red-winged blackbird T_4_ plasma concentration similarly peaks around 4 dph and remains elevated through 10 dph. These studies in altricial species, as well as those concerned with precocial species of birds (Sirsat and Dzialowski, 2020), suggest that functional maturation of the HPT axis is critical to the ontogeny of endothermic thermoregulatory development in birds (Olson et al., 1999). Nonetheless, only a few studies have manipulated thyroid hormones during the nestling period to test this hypothesis.

In the present study, we investigated how altering plasma T_3_ levels during the first 5 days of post-hatching life influenced development and growth of red-winged blackbird nestlings up to fledging. We pharmacologically altered T_3_ plasma levels during the first 5 days of post-hatching life to manipulate the timing of the increase in plasma T_3_ levels in relation to the normal developmental trajectory of plasma T_3_ levels (Olson et al., 1999). This manipulation allowed us to examine the role of circulating plasma T_3_ on nestling growth and development of endothermic capacity. Additionally, we measured how development of ventilatory control associated with the cold-induced metabolic response responded to altered thyroid hormone levels. We predicted that in response to early nestling hypothyroid conditions the development of endothermy, measured as a strong whole animal metabolic rate and ventilation response to cooling, would occur later in the nestling period. In contrast, we expected nestling hyperthyroid conditions to accelerate the development of endothermy to earlier in the nestling development.

## Materials and methods

### Animals and collection sites

Red-winged blackbird nestlings were collected between May and July of 2014 and 2015 from the Lewisville Aquatic Ecosystem Research Facility (LAERF) and two retention ponds on the University of North Texas campus in Denton County in north Texas (USFWS Permit No. MB02732B-2, TPWS permit #SPR-0214-034 to E. M. Dzialowski). Nests were located and monitored daily in the morning or early afternoon to determine timing of egg laying and hatching sequence and to assess nestling status throughout the nesting period. Sample sizes were determined by the number of successful nests found in our study population.

### Experimental design and drug administration

Experiments were conducted over two consecutive years. During the first year, nestlings were orally treated with either methimazole (MMI), a thyroperoxidase inhibitor, or water as a control. During the second year, nestlings were orally treated with supplemental 3,3’, 5- Triiodo-L- thyronine (T_3_) or water as control. We treat each year as a separate experiment.

Each year, nests containing at least three eggs were randomly assigned into one of the two groups. In 2014, nests were assigned either a hypothyroid treatment (10 mg MMI/kg nestling mass, MP Biomedicals, Solon, Ohio) or control (equal volume of water as of the methimazole treatment). In 2015, nests were assigned either a hyperthyroid treatment (5 mg T_3_/kg nestling mass, Sigma-Aldrich, St Louis, MO, United States) or control (equal volume of water as of the T_3_ treatment). The T_3_ solution was prepared by dissolving 3,3’, 5- Triiodo-L- thyronine in sterile 0.2M NaOH and diluted with 0.9% NaCl sterile saline. The MMI solution was prepared by dissolving methimazole in 0.9% NaCl sterile saline. Nestlings were left in their nest and raised and fed by their parents in the nest until the day they were to be exposed to the cold challenge in the lab. In the field, nestlings were weighed daily after hatching and received an oral dose with a volume in microliters equal to their mass in grams by Gilson pipette of their respective treatments on 2-, 3- and 5-day post-hatch (dph).

Nestlings on 5, 7, or 9 dph were collected from their nest in the field in the morning and transported to the University of North Texas (32 km) and used within 2–4 h of arrival in the laboratory. Nestlings were kept in a bench top incubator at 35°C (model 1602N, Hova-bator G.Q.F. manufacturing, Savannah, GA) and fed roach nymphs (*Blaptica dubia*) *ad libitum* until experiments began.

A subset of nestlings were brought to the laboratory on 4 dph, dosed with either T_3_ or saline solution, and blood samples were collected at 2, 8, 12, 18 and 24 h intervals after drug administration to determine the rate of plasma T_3_ metabolism after T_3_ supplementation.

### Whole-animal metabolic rate and body temperature

Oxygen consumption rate ( ${\dot{V}o}_{2}$ ), a proxy of metabolic rate, was measured using flow-through respirometry during gradual cooling as in Sirsat and Dzialowski (2020). Animals were placed in respirometry chambers (∼250 and 500 ml) which were placed inside a programmable, insulated incubation cabinet set to 35°C. Animals were acclimated for at least 60 min prior to beginning of a gradual cooling protocol. The incubation cabinet temperature was then gradually decreased at a rate of 10°C h^−1^ until the ambient temperature (T_a_) reached 15°C. Gaseous mixture between 20.9% and 21.3% oxygen balanced with nitrogen was mixed by a microprocessor control unit (controller model 0154, Brooks Instruments, Hatfield, PA) and flow controllers (model 5850E, Brooks Instruments, Hatfield, PA) then pumped into each respirometry chamber at a known flow rate between 140 and 150 ml min^−1^. Flow rate was measured using a calibrated FlowBar1 mass flow meter (Sable Systems International, Las Vegas, NV). A portion of outflow gas from the respirometry chamber was pulled by a pump (R1 flow controller, AEI Technologies, Pittsburgh, PA) through Nafion tube (ADInstruments, Colorado Springs, CO) covered in drierite, a column of Sodasorb, and another Nafion tube for water and CO_2_ removal, before it was pulled through an O_2_ analyzer (FC-1B, Sable Systems). Sampling of outflow gas from up to 3 respirometry chambers was automatically controlled by a custom-built solenoid multiplexer controlled by LabChart 7 (ADInstruments). A maximum of 3 animals were measured at any given time and each chamber was sequentially sampled for 150 s. An extra solenoid was used to sample the inflow O_2_ level, allowing inflow gas to be sampled every 7.5 min. Data were recorded with PowerLab 16SP and LabChart 7 software (ADInstruments). Rates of oxygen consumption ( ${\dot{V}o}_{2}$ ml O_2_ min^−1^) were calculated using the following equation (Withers, 2001).

$${\dot{V}o}_{2}={\dot{V}}_{I}\times \frac{\left(F_{I}o_{2}-F_{E}o_{2}\right)}{1-F_{E}o_{2}}$$ (1)

Where ${\dot{V}}_{I}$ is incurrent flow rate (ml min^−1^), F_I_o_2_ is the incurrent O_2_ fraction of dry gas, and F_E_o_2_ is the excurrent O_2_ fraction of dry gas.

Along with measuring ${\dot{V}o}_{2}$ , animal cloacal body temperature (T_b_) was continuously recorded. A thermocouple was placed in the cloaca and secured with the help of a small plastic disk glued to the feathers of the hatchling with super glue (Loctite^®^, Henkel Corp. Westlake, Ohio) as in Sirsat and Dzialowski (2020). T_b_ was measured using ADinstruments temperature pods and recorded with a PowerLab 16SP and LabChart 7 (ADInstruments). In 2014 in 3 MMI nestlings and 1 control nestling, the T_b_ signal dropped out once the cooling experiment began and did not return resulting in no temperature recorded for those animals. In an additional 4 MMI nestlings and 1 control nestling, the T_b_ signal would intermittently drop out and come back during the cooling experiment. For these animals with intermittent missing T_b_, we used non-linear interpolation using the cooling T_b_ from that animal across the cooling run to estimate the missing T_b_.

### Pulmonary ventilation

Estimates of tidal volumes in the thermoneutral zone and during cooling were made using a barometric technique described in Sirsat and Dzialowski (2016), Sirsat and Dzialowski (2020). Changes in pressure associated with breathing were measured with a spirometer (ADIstruments) connected in-line with each respirometry chamber (Menna and Mortola, 2003). Volume calibration was conducted in each respirometry chamber after each trial by injecting known volumes of air (Vcal) into the system by Hamilton syringe (Hamilton, Reno, NV). The corresponding change in pressure (Pcal) was used to calibrate the system (K = Vcal/Pcal as in (Szdzuy and Mortola, 2008)). Each respirometry chamber’s relative humidity was measured using humidity sensors (HIH 4021, Honeywell, Minneapolis, MN) and included to estimate chamber water vapor pressure. Tidal volume estimates were calculated from K, chamber temperature, T_b_, water vapor pressure, and measured pressure changes. Breathing frequency (ƒ, breaths/min) was recorded from the pressure waves using the cycle measurement function in LabChart 7 software (ADInstruments). Minute ventilation (ml min^−1^) was calculated by multiplying tidal volume by ventilation frequency. For two 7 dph nestlings in 2014 (one control and one MMI), the temperature probe did not function resulting in no body temperature data for calculation of tidal volume. For these two 7 dph nestlings with missing T_b_, we carried out mean imputations of T_b_ using the T_b_ from 7 dph nestlings within the given treatment to calculate tidal volume and minute ventilation.

### Blood collection and morphometrics

After metabolic rate and pulmonary ventilation measurements, the animals were anesthetized with isoflurane. Blood (approximately 0.5–1 ml) was collected from anesthetized animals in heparinized 1 ml plastic syringes from the atria of the heart by cardiac puncture. Blood was immediately transferred into a 1.5 ml plastic vial and centrifuged (Model 16K, Bio-Rad, Hercules, CA) at 3,000 rpm for 10 min at 4°C. Plasma was aliquoted and stored at −20°C for later plasma T_3_ analysis. Animals were euthanized by decapitation under isoflurane anesthesia and dissected. Wet masses of whole body, yolk mass, cardiac ventricles, liver, heart, both kidneys, and both lungs were measured (±0.1mg, Item no. E12140, Ohaus Corp., Pine brook, NJ). To examine structural size, lengths of head to beak (from the posterior end of the supraoccipital bone to the tip of the beak, with the skin intact), femur, tarsus, and wing cord were measured using calipers (±0.01 mm Traceable^®^ Fisher Scientific, Pittsburgh, PA).

Total plasma T_3_ concentrations were measured in duplicate as in Sirsat and Dzialowski (2020), using an AccuBind T3 ELISA (125-300 Monobind Inc., Lake Forest, CA, United States).

### Data analysis

We examined responses using a measure of standardized effect size, Cohen’s d with 95% confidence interval, because we were interested in the magnitude of the effect of altering plasma T_3_ hormone status on the various measured parameters within each nestling age (Nakagawa and Cuthill, 2007; Sneddon et al., 2017). When assessing responses to altering T_3_ levels with Cohen’s d effect sizes we used the following ranges for small (0.49 > d > 0.2), moderate (0.79 > d > 0.5), large (1.19 > d > 0.8), or very large (>1.2) effects. Body mass was analyzed by ANOVA with age and treatment as factors. We used an ANCOVA to obtain estimated marginal means accounting for body mass as a covariate of heart ventricle, liver, lungs, and kidney and lengths of the femur, tarsus, wing cord, and head-to-beak. Body mass was included as a covariate to account for differences in body masses between treatments. For all morphological measures, the effect size was determined for the control compared with the treated response at each age as Cohen’s d ± 95% CI from the ANCOVA estimated marginal means (Nakagawa and Cuthill, 2007). Morphometry data is presented as the ANCOVA estimated marginal means ±95% CI accounting for mass as the covariate. Oxygen consumption, ventilation frequency, tidal volume, minute ventilation, and T_b_ were all analyzed by two-way repeated measured ANOVA with thyroid treatment and ambient temperature as factors. When ambient temperature was significant in the model, we separated the data by treatment to determine the ambient temperature at which differences first appeared in the treatments (Dzal and Milsom, 2021). We did this by looking at paired Cohen’s d effect sizes ±95% CI to determine at what temperatures there was a large or greater effect (>0.8) of the treatment on the parameter in question. We considered Cohen’s d values of importance when their ±95% CI did not encompass 0. Statistical analyses were performed in Jamovi running R version 4.0.2 (https://www.jamovi.org/) and Prism 9.

## Results

### Plasma T_3_

**FIGURE 1:**
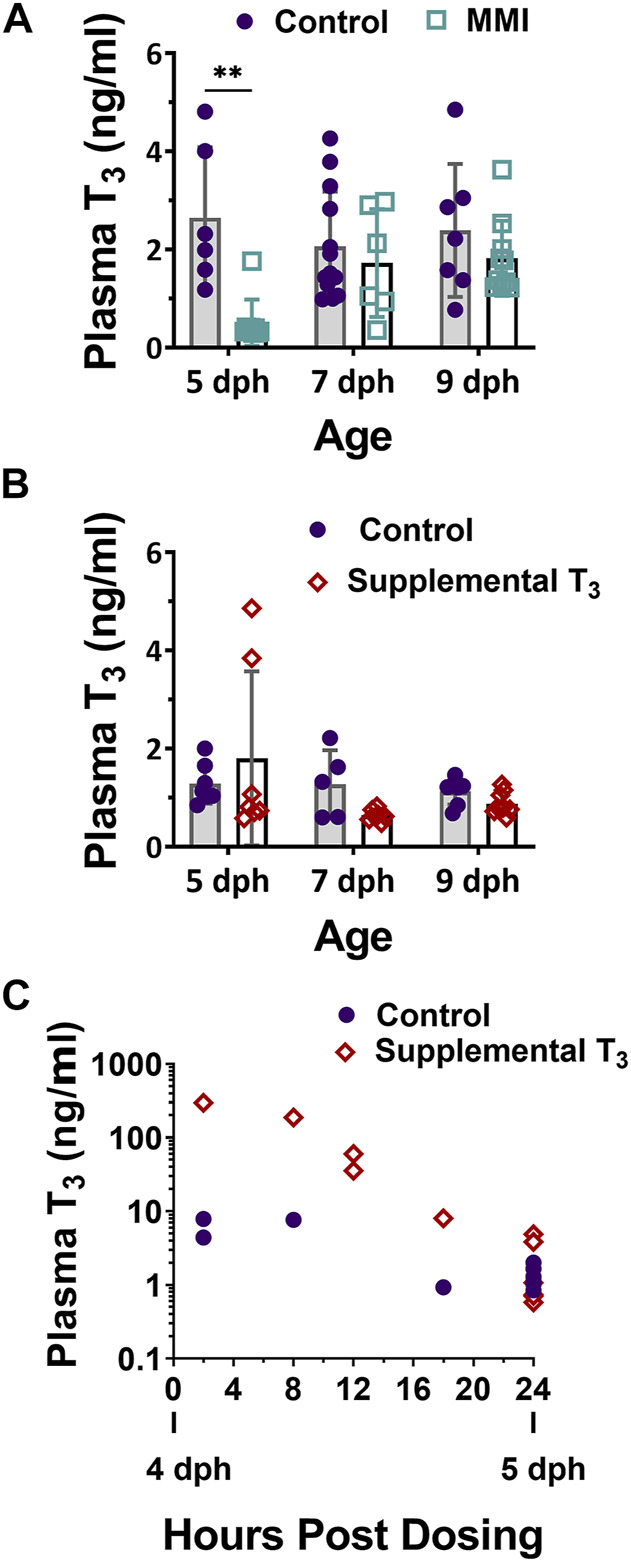
Plasma 3,3′, 5- Triiodo-L- thyronine (T_3_) levels in 5, 7, and 9 dph red-winged blackbird nestlings after **(A)** Methimazole (MMI) or **(B)** T_3_ treatment on 2, 3, and 5 dph. **(A)** After treatment with MMI, plasma T_3_ levels were lower than controls but recovered to control levels by 9 dph. Control n—5dph = 6, 7 dph = 13, 9 dph = 7; MMI n—5 dph = 9, 7 dph = 6, 9 dph = 10. **(B)** Treatment with T_3_ did not produce a large effect in plasma T_3_ levels at any age. Contol n—5 dph = 7; 7 dph = 5; 9 dph = 7; T_3_ n—5 dph = 7; 7 dph = 7; 9 dph = 9. **(C)** 4 dph nestling plasma T_3_ increased within 2-h of oral T_3_ dosing and returned to control levels within 24-h after oral dosing. Data presented as mean ± standard deviation. ** indicates significant difference between control and treatment within an age at *p* < 0.001 and a very large Cohen’s d effect size.

**FIGURE 2:**
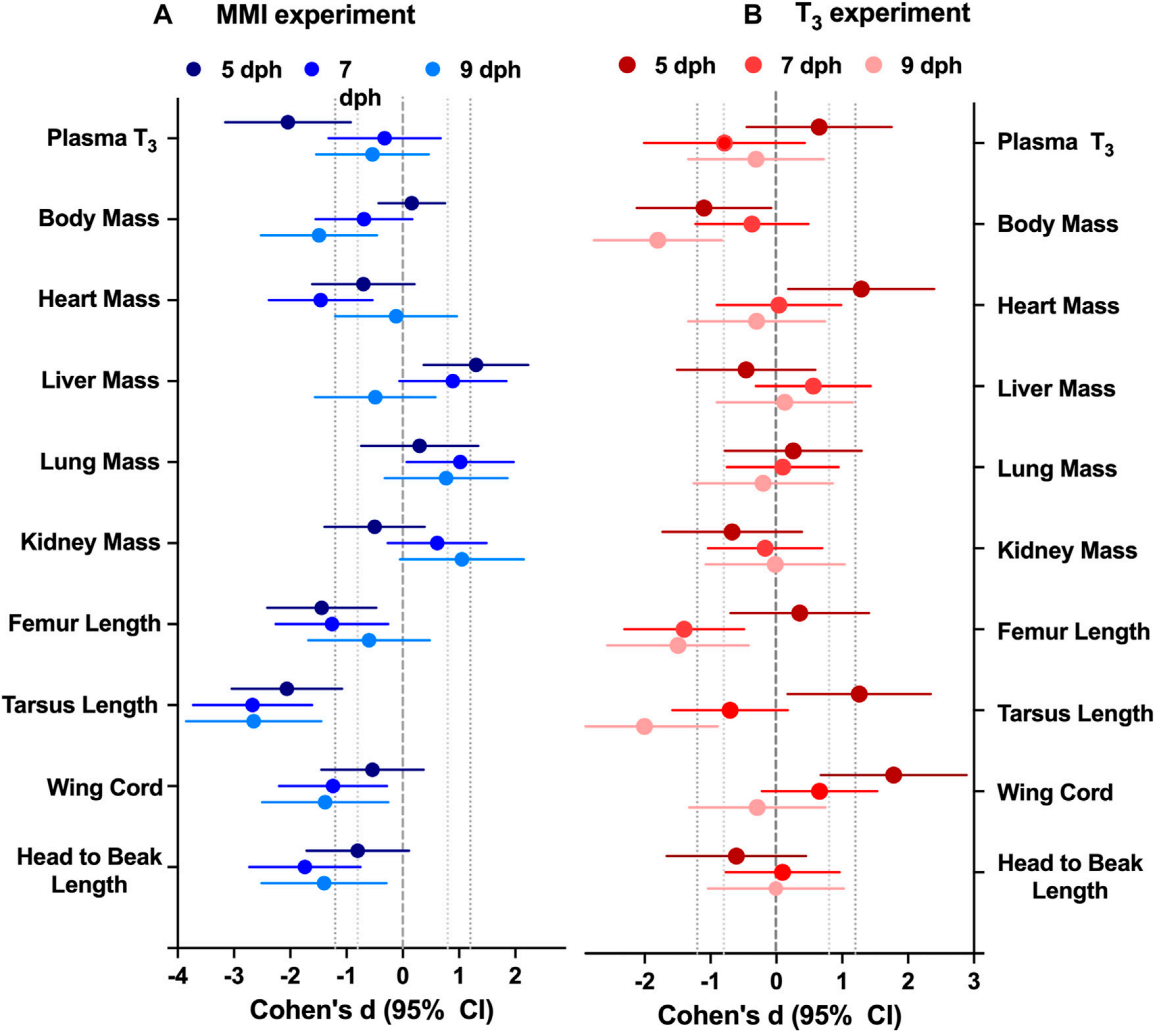
Effect of altering thyroid plasma levels during the first half of nestling period on morphology differed between **(A)** hypothyroid and **(B)** hyperthyroid treatments. Data presented as the effect size estimate Cohen’s d ± 95% confidence intervals comparing the effect of the treatment with control values within an experimental year. Cohen’s d effect size are calculated from the ANCOVA estimated marginal means with body mass as a covariate to remove the effect of body mass. n = 7–10 per treatment.

Treatment with MMI on days 2, 3 and 5 post-hatch had a large effect on the plasma T_3_ levels of nestlings (Figure 1A, Figure 2A; treatment effect F_1, 45_ = 11.06, *p* = 0.001). On day 5 post-hatch, MMI treatment during the first half of the nestling period had a large negative effect on the nestling’s plasma T_3_ levels that disappeared by 9 dph (Figure 2A).

Treatment of nestlings with T_3_ on days 2, 3, and 5 post-hatch had little effect on the nestling plasma T_3_ levels on 5, 7 and 9 dph (Figure 1B, Figure 2B; treatment effect F_1, 39_ = 0.01, *p* = 0.091). There was a moderate elevated effect on 5 dph plasma levels in some animals, although samples were quite variable. Nestlings that had received supplemental T_3_, trended lower plasma T_3_ levels than controls on 7 dph, but were similar to controls on 9 dph. To ensure our method of supplementing T_3_ led to an elevation in plasma T_3_ during the first half of the nestling period, additional animals were dosed on 4 dph and their plasma T_3_ levels measured at 2, 8, 12, 18, and 24 h post-dosing (Figure 1C). At 2 h, plasma T_3_ levels were elevated above the controls and returned to control levels by 24 h post exposure.

### Morphology

Treatment with MMI produced a very large effect on the growth trajectories of the morphological parameters examined (Figure 2; Table 1). Treatment with MMI had a very large negative effect on body mass on 9 dph, 4 days after the last dose of MMI. Induction of early hypothyroidism had a very large negative effect on heart mass and large positive effects on liver, lung, and kidney mass (Figure 2A). Structurally, exposure to MMI had a very large negative effect on size of the nestlings (Figure 2A). MMI treated nestlings tended to have shorter femurs, tarsus, wing cord, and head to beak length than controls (Figure 2A).

**TABLE 1:** Nestling red-winged blackbird body masses and ANCOVA estimated marginal means of morphometric measures with body mass as a covariate from MMI treatment experiment and T_3_ treatment experiment. Data presented as mean [95% CI]. Values in bold with an asterisks indicate very large effects (Cohen’s d > 1.2) of treatment on the parameter in comparison to the control.

	Age	Control	MMI	Control	T_3_
N	5 dph	10	10	7	8
	7 dph	14	8	9	12
	9 dph	7	10	9	11
Body mass (g)	5 dph	18.8 [16.2, 21.3]	19.4 [16.9, 21.9]	21.0 [18.5, 23.4]	17.1 [14.6, 19.6]*
	7 dph	27.2 [25.1, 29.4]	24.5 [21.8, 27.2]	23.3 [21.1, 25.6]	22.1 [20.1, 24.1]
	9 dph	30.1 [21.7, 33.2]	24.2 [21.7, 26.7]*	29.5 [27.1, 31.8]	23.2 [21.1, 25.3]*
Heart mass (mg)	5 dph	190 [172, 207]	173 [156, 190]	169 [155, 184]	193 [177, 208]*
	7 dph	188 [174, 202]	154 [138, 170]	172 [160, 184]	173 [162, 183]
	9 dph	185 [165, 206]	182 [167, 197]	174 [158, 189]	168 [157, 179]
Liver (mg)	5 dph	998 [893, 1102]	1183 [1082, 1285]*	937 [856, 1018]	885 [791, 980]
	7 dph	926 [842, 1010]	1053 [952, 1155]	879 [808, 950]	942 [877, 1007]
	9 dph	948 [822, 1074]	879 [789, 970]	811 [716, 907]	826 [758, 893]
Lung (mg)	5 dph	199 [172, 227]	226 [200, 253]	197 [183, 211]	192 [176, 208]
	7 dph	210 [188, 233]	249 [222, 275]	178 [165, 190]	179 [168, 191]
	9 dph	221 [188, 254]	250 [226, 273]	187 [170, 204]	183 [172, 195
Kidney (mg)	5 dph	396 [367, 425]	394 [365, 422]	424 [394, 454]	396 [361, 431]
	7 dph	394 [370, 417]	418 [390, 447]	379 [352, 405]	371 [347, 395]
	9 dph	376 [341, 412]	418 [393, 444]*	367 [330, 404]	367 [341, 392]
Tarsus (mm)	5 dph	24.8 [24.2, 25.5]	23.0 [22.4, 23.6]*	23.4 [22.7, 24.0]	24.5 [23.8, 25.3]*
	7 dph	26.4 [25.8, 26.9]	24.0 [23.3, 24.6]*	25.7 [25.2, 26.3]	25.1 [24.6, 25.6]
	9 dph	27.8 [27.0, 28.6]	25.4 [24.8, 26.0]*	27.3 [26.5, 28.1]	25.5 [24.9, 26.0]*
Femur (mm)	5 dph	23.3 [22.6, 24.0]	21.8 [21.1, 22.6]*	22.3 [21.8, 22.9]	22.6 [22.0, 23.2]
	7 dph	23.7 [23.1, 24.3]	22.4 [21.7, 23.2]*	22.7 [22.2, 23.2]	21.6 [21.2, 22.1]*
	9 dph	23.3 [22.5, 24.2]	22.7 [22.1, 23.4]	22.8 [22.2, 23.5]	21.7 [21.2, 22.1]*
Wing cord (mm)	5 dph	39.0 [36.6, 41.3]	37.2 [34.9, 39.5]	35.8 [34.0, 37.6]	40.2 [38.1, 42.3]*
	7 dph	48.3 [46.4, 50.2]	44.3 [42.0, 46.5]*	47.8 [46.2, 49.4]	49.4 [48.0, 50.9]
	9 dph	57.2 [54.3, 60.0]	52.7 [50.7, 54.8]*	57.4 [55.3, 59.6]	56.7 [55.2, 58.2]
Head to Beak (mm)	5 dph	27.7 [26.7, 28.8]	26.6 [25.6, 27.6]	27.8 [25.2, 30.3]	25.6 [22.6, 28.6]
	7 dph	29.1 [28.3, 30.0]	26.6 [25.6, 27.7]*	29.0 [26.8, 31.3]	29.4 [27.3, 31.4]
	9 dph	30.5 [27.6, 29.4]	28.5 [27.6, 29.4]*	30.5 [27.4, 33.5]	30.4 [28.3, 32.6]

Treatment with supplemental T_3_ had a large to very large negative effect on body mass of 5 and 9 dph nestlings compared with controls (Figure 2B; Table 1). Initially, on 5 dph, there was a very large positive effect of supplemental T_3_ on heart mass that disappeared by 7 dph. There was little effect of supplemental T_3_ on the other organ masses on 5, 7 and 9 dph (Figure 2). Structurally, T_3_ treatment initially had very large positive effect on tarsus length and wing cord compared with controls. By 9 dph the effect of supplemental T_3_ became a very large negative effect on tarsus length and no effect on wing cord lengths. Supplemental T_3_ also had a very large negative effect on femur length on 7 and 9 dph.

### Oxygen consumption

**FIGURE 3:**
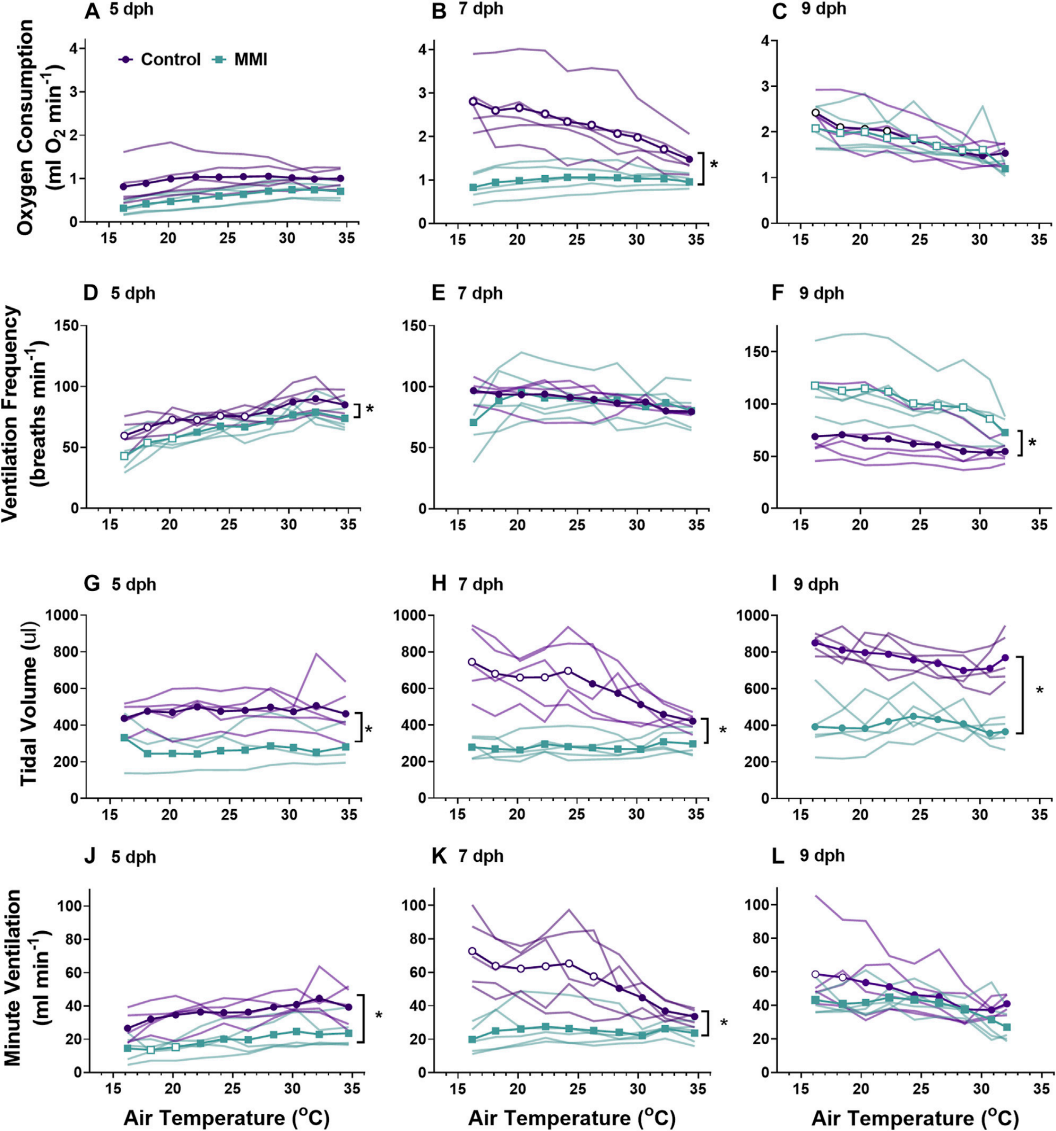
The ontogeny of oxygen consumption **(A–C)**, ventilation frequency **(D–F)**, tidal volume **(G–I)**, and minute ventilation **(I–K)** were affected by treatment with MMI. Data presented as means and individual responses. * indicates a significant treatment effect at *p* < 0.05. Within a treatment, open symbols represent large or greater effects of temperature based on Cohen’s d effect size >0.8 compared with the thermal neutral value. n = 5 per treatment.

**FIGURE 4:**
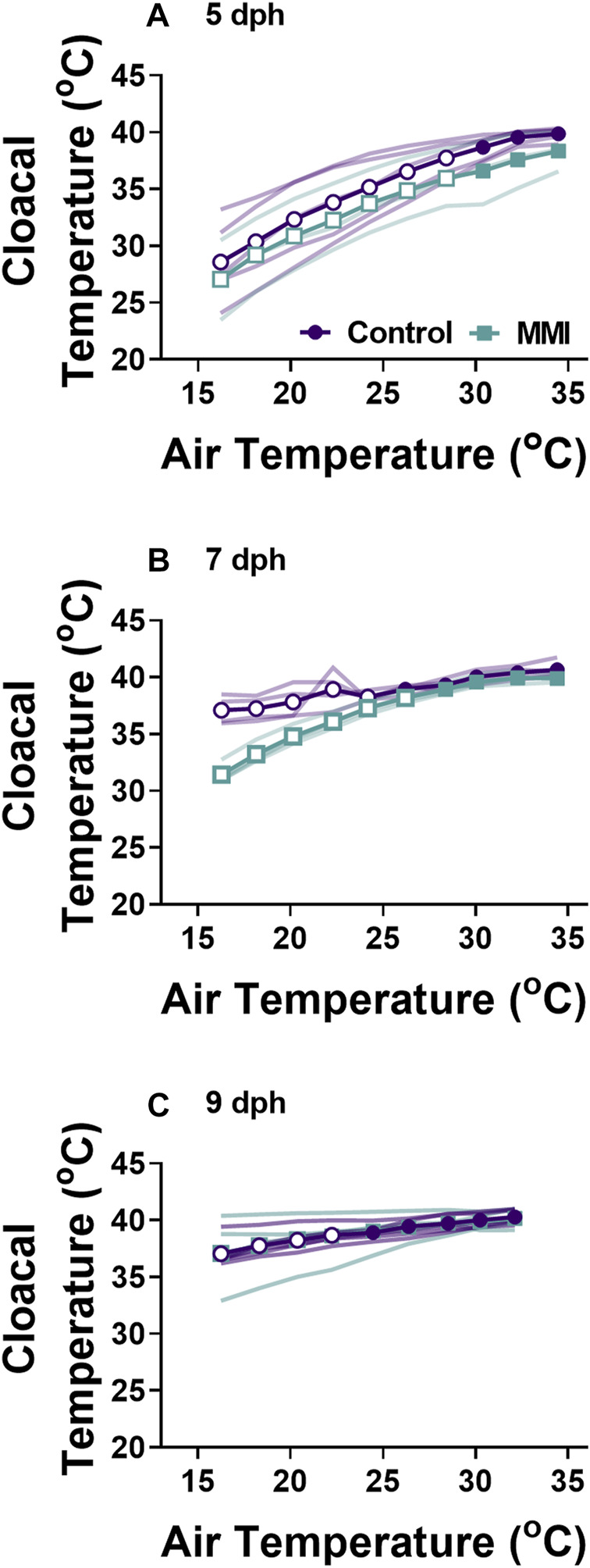
Changes in body temperature during cooling for control and MMI treated nestlings. Data presented as means individual responses. Within a treatment, open symbols represent large effect of temperature based on Cohen’s d effect sizes >0.8 compared with the thermal neutral value. n = 5 for 5 dph control and 9 dph control and MMI, n = 3 for 5 dph MMI, and n = 4 for control and MMI 7 dph.

Treatment with MMI resulted in altered developmental trajectories in the response of ${\dot{V}o}_{2}$ to decreasing ambient temperature (Figures 3A–C). Oxygen consumption rate of MMI treated and control nestlings responded similarly to decreasing ambient temperature on 5 and 9 dph, but nestling responses from the two treatments deviated on 7 dph (Figure 3A). In 5 dph nestlings, the ${\dot{V}o}_{2}$ response to cooling was minimal (all Cohen’s d < 0.5). ${\dot{V}o}_{2}$ responded differently in 7 dph nestling (treatment effect F_1.09, 8.733_ = 10.28, *p* = 0.010). In 7 dph nestlings during cooling, there was no change in ${\dot{V}o}_{2}$ in MMI treated nestlings at all temperatures tested compared with control nestlings that exhibited a very large increase in ${\dot{V}o}_{2}$ beginning at an ambient temperature of 32°C (Cohen’s d > 1.2; Figure 3B). By 9 dph, control and MMI treated nestling ${\dot{V}o}_{2}$ exhibited very large increases in response to decreasing ambient temperatures at 22°C and 30°C respectively (Figure 2C; Cohen’s d > 1.0). Cloacal temperatures only differed between MMI treated and control nestlings on 7 dph (Figure 4; treatment effect F_1,6_ = 31.41, *p* = 0.0014).

**FIGURE 5:**
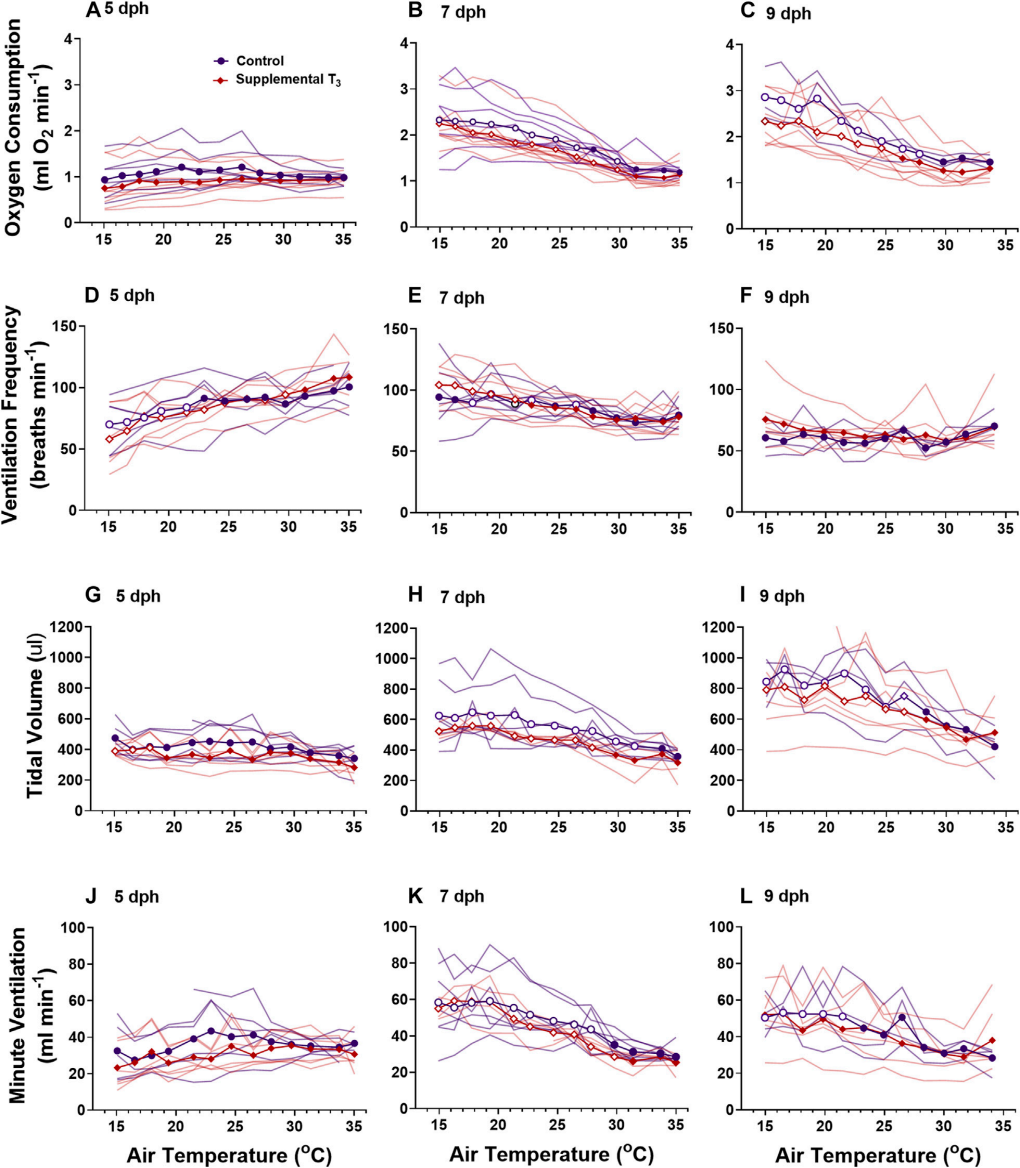
The ontogeny of oxygen consumption **(A–C)**, ventilation frequency **(D–F)**, tidal volume **(G–I)**, and minute ventilation **(I–K)** were not affected by supplemental T_3_. There was no effect of T_3_ treatment on metabolism or ventilation. Data presented as means plus individual responses. Within a treatment, open symbols represent large or greater effects of temperature based on Cohen’s d effect sizes >0.8 compared with the thermal neutral value. n = 5 per treatment.

Oxygen consumption rates from control and supplemental T_3_ nestlings followed the same developmental trajectories (Figures 5A–C). There was no change in ${\dot{V}o}_{2}$ in response to decreasing ambient temperature in 5 dph nestlings from either treatment (Figure 5A). Nestlings from both treatments on 7 and 9 dph exhibited a thermal neutral zone between 35 and 31°C where ${\dot{V}o}_{2}$ was constant (Figures 5B,C). At ambient temperatures below 30, ${\dot{V}o}_{2}$ increased in a linear fashion for both ages and treatments. There were no differences in cloacal temperature between control and supplemental T_3_ nestlings on any day (data not shown).

### Ventilation

We observed differential responses to cooling in ventilation parameters based on age and MMI treatment (Figure 3). On day 5 post-hatching, control nestlings’ ventilation frequency (Figure 3D; F_1, 8_ = 13.45, *p* = 0.0063), tidal volume (Figure 3G; F_1,8_ = 9.20, *p* = 0.0162), and minute ventilation (Figure 3J; F_1,8_ = 19.44, *p* = 0.0023) were all larger than those of MMI treated nestlings, but the response to cooling was similar between the two treatments. Comparing 7 dph control and MMI treated nestlings, there were treatment effects on tidal volume (Figure 3H; F_1,8_ = 25.8, *p* = 0.001) and minute ventilation (Figure 3K; F_1,8_ = 14.73, *p* = 0.005) with control nestling levels being higher than MMI treated nestlings. In control 7 dph nestlings, the very large increase in minute ventilation associated with the decreasing ambient temperature was accomplished by a very large increase in tidal volume with no change in ventilation frequency (Figures 3E,H,K). Minute ventilation, tidal volume and ventilation frequency remained unchanged during cooling in the 7 dph MMI treated nestlings. In 9 dph nestlings, both control and MMI treated nestlings increased minute ventilation but did so in different ways (Figures 3F,I,L). Control nestlings had a higher tidal volume than MMI treated nestlings (Figure 3I; F_1, 8_ = 80.0, *p* < 0.0001) and small increases in both tidal volume and frequency combined to increase minute ventilation (Figures 3F,I; Cohen’s d < 0.8) while MMI treated nestlings had a smaller tidal volume than controls and had very large increases in ventilation frequency in response to cooling (Figure 3F).

Supplementing nestlings with T_3_ early in the nestling period had no effect on the development of ventilation or ventilatory responses to cooling at any age (Figure 5).

## Discussion

The major question addressed in this study was whether altering thyroid hormone levels during the first 5 days post hatching would influence the growth and metabolic developmental trajectories of altricial red-winged blackbird nestlings from day 5 through 9 dph. Collectively, our data indicate that circulating thyroid hormones are important regulators of the development of endothermy and pulmonary ventilation in this species. Larger developmental effects in both morphology and physiology were observed in response to a hypothyroid condition when compared with the responses in control and hyperthyroid conditions.

### Plasma T_3_ levels

Altricial nestlings exhibit a rise in plasma T_3_ levels during the first few days of post-hatching life followed by a plateau and then decline to adult levels by fledging (McNabb and Cheng, 1985; McNabb, 2006; De Groef et al., 2013). In red-winged blackbird nestlings, plasma T_3_ levels initially rose at hatching, plateaued around 4 dph, and remained elevated through 10 dph (Olson et al., 1999). Similar plasma T_3_ developmental trajectories have been observed in ring doves (McNabb and Cheng, 1985), European starlings (Schew et al., 1996; Výboh et al., 1996), great tit (Silverin and Rudas, 1996), zebra finch (Yamaguchi et al., 2017), and semi-altricial king penguin (Cherel et al., 2004). On day 5 post-hatching, plasma T_3_ levels in control nestlings appear to have reached the plateau as observed in other altricial and semi-altricial hatchlings (Figure 1).

In both years’ experiments, nestlings were dosed on days 2, 3, and 5 post-hatching in order to alter the natural developmental trajectory of plasma T_3_ levels. In 2014, treatment with MMI on 2, 3, and 5 dph resulted in a large decrease in plasma T_3_ levels on 5 dph compared to the control (Figure 1A). MMI primarily accumulates in the thyroid gland and lowers the production of T_4_ by inhibiting thyroid peroxidase (Vickers et al., 2012; Herck et al., 2013). Although plasma T_4_ was not measured in the current study, we expect plasma T_4_ decreased leading to a decreased production of T_3_ and the subsequent lower plasma levels of T_3_ in 5 dph MMI treated group. After cessation of MMI administration on 5 dph, plasma T_3_ levels began to increase by 7 dph. The 7 dph and older nestlings in the current study were presumably able to elevate T_3_ levels because of the eventual metabolism of MMI and subsequent production of T_4_ and T_3_.

Based on T_3_ plasma levels measured on 5 dph, supplemental T_3_ had little effect on nestling plasma T_3_ levels. However, upon halting the supplemental T_3_ after 5 dph, there was a trend for plasma T_3_ levels to be lower on 7 dph in supplemented vs. control nestlings which suggests that elevation of plasma T_3_ on 2, 3, and 5 dph may have inhibited the development of the thyroid gland. This lower T_3_ after removal of supplemental T_3_ may be due to reflex inhibition by exogenous T_3_ on the hypothalamic pituitary thyroid axis (McNabb, 2007). We expect that the supplemental T_3_ elevated plasma T_3_ on days 2, 3, and 5 dph as we found with our time course in the 4 dph nestlings. Since the 5 dph animals in which plasma T_3_ was measured were not dosed in the 18 h prior to their plasma being sampled (Figure 1C), we did not observe an increase in their plasma T_3_ at the 5 dph timepoint. In nestlings dosed on 5 dph in the field, their plasma levels were expected to have risen upon dosing and fallen back to control levels by 6 dph. The rapid elevation of plasma T_3_ followed over the next 24 h by a rapid decrease down to control group’s plasma levels (Figure 1C) suggests that nestlings rapidly metabolized the supplemental T_3_, allowing for rapid plasma clearance. The rate of T_3_ metabolism is not surprising, given this species has one of the highest postnatal growth rates recorded in birds (Olson, 1992).

### Morphology

Altering the plasma T_3_ trajectory during the first 5 days of post-hatch life altered nestling growth. Newborn altricial red-winged blackbird hatchlings allocate energy towards rapid growth during the first week of nestling development before they develop proficient endothermic capacity towards the end of nestling period (Olson, 1992). In Texas, we found our nestlings fledge rapidly within 10 days of hatching. The response of nestling morphology on days 5–9 was altered by both hyperthyroid and hypothyroid conditions during the first 5 days as a nestling. The T_3_ hyperthyroid treatment resulted in significant decrease in body mass at 5 and 9 dph (Figure 2). Similarly, the MMI hypothyroid treatment resulted in significant decrease in nestling body mass at 7 and 9 dph, compared to control groups in red-winged blackbirds. Body mass did not differ between 5 dph MMI hypothyroid nestlings and control nestlings, but MMI treated nestling body mass began to lag behind controls after 5 dph. Similar differences in body mass have been observed previously where pharmacologically inducing hypothyroidism decreased body mass in altricial zebra finch nestlings (Rainwater et al., 2008), precocial Barnacle geese (Deaton et al., 1998) and broiler chicken (Decuypere et al., 1987) hatchlings. Prolonged treatment with 6-n- propyl-2-thiouracil (PTU), another thyroperoxidase inhibitor with similar action to that of MMI, from 1 to 5 weeks after hatch, resulted in hindered growth of Muscovy ducklings (Rey et al., 2010). These observations of slower growth both in hyper- and hypothyroidism indicate the need for maintaining optimum levels of plasma T_3_ levels during the rapid growth period in red-winged blackbird nestlings.

Along with mass, structural size of the nestlings was influenced by the thyroid plasma levels experienced during the first 5 days of the nestling phase. Hypothyroid conditions during the first 5 dph resulted in decreased structural size compared with controls (Figure 2). Similar decreases in structural size were observed in Zebra finch nestlings treated with perchlorate, an iodine transport inhibitor (Rainwater et al., 2008). In T_3_ supplemented nestlings, structural sizes did not begin to decrease compared with controls until after 5 dph when the T_3_ levels were dropping in the hyperthyroid nestlings. Hypothyroidism decreased tibiotarsal length in altricial zebra finch nestlings possibly due to growth plate dysgenesis as observed in mammals (Harvey et al., 2002; Rainwater et al., 2008). It is unclear if these differences in structural size would translate into differences in flight capacity of adults.

Hypothyroidism and hyperthyroidism differentially influenced the growth of tissues such as the heart and liver within the nestling. Hearts from MMI treated nestlings were smaller on 5 and 7 dph compared with the controls; the same organs were larger in T_3_ treated nestlings at 5 dph. Cardiac mass of 1 dph (Sirsat and Dzialowski, 2020) and 8 weeks Pekin duck (Bishop et al., 2000) were similarly decreased by MMI treatment. Deaton et al. (1998) also reported lower relative ventricle mass in hypothyroid barnacle geese and suggested the effect could be due to reduction of cardiac protein synthesis resulting in lower cardiac muscle mass. Hearts from 1 dph Pekin duck treated with T_3_ during late incubation were not different in mass from the controls either during external pipping or 1 dph (Sirsat and Dzialowski, 2020).

Liver mass was elevated in the MMI treated nestlings on days 5 and 7 post hatching and returned to control levels by 9 dph (Figure 2). Similar elevations in liver mass were observed in Zebra finch nestlings that had been treated with the perchlorate (Rainwater et al., 2008), pekin duck embryos (Sirsat and Dzialowski, 2020), and broiler chicken hatchlings (Decuypere et al., 1987) treated with MMI. Treatment with MMI in humans has been shown to produce hepatotoxicity (Gomez-Peralta et al., 2018). This increase in liver mass may be in response to the hepatotoxic MMI.

### Whole-animal metabolic rate and body temperature

Red wing blackbird nestlings develop an endothermic response to gradual cooling around 7 dph (Olson, 1992). Prior to this time, they are able to maintain metabolic rate, but not elevate it. Tazawa et al. (2001) suggested that the maturation of an endothermic response to cooling correlated with the rise in thyroid hormones in developing birds. In the red-winged blackbird, T_3_ levels rise and plateau around 4-5 dph with plasma T_4_ levels peaking on 7 dph and both remain elevated through 9 dph (Figure 1A, (Olson et al., 1999)). Associated with this T_3_ plateau is the rise of an incipient endothermy where metabolic rate does not increase but is maintained over a relatively wide range of temperatures in 5 dph nestlings (Figure 3A; (Olson, 1992)), with a strong endothermic response by 7 dph (Figure 3B). The hyperthyroid nestlings show a similar endothermic developmental trajectory as control nestlings. Development of a strong endothermic response of hypothyroid nestlings was delayed compared with euthyroid nestlings (Figures 3A–H). This delay correlates well with the trajectory of plasma T_3_, being low in 5 dph nestlings and rising to a plateau by 7 dph (Figure 1A). Previous studies on nestling altricial birds observed that growth of the thyroid gland is highest just before the development of endothermy (Olson et al., 1999; McNabb, 2006). In nestling starlings, thyroid hormones plateau around 10 dph (McNabb, 2006) with the strongest endothermic metabolic response to cooling beginning on 12 dph (Clark, 1982). A similar relationship between thyroid hormone levels and the development of endothermy have been observed in a marsupial, the Tasmanian bettong (Rose and Kuswanti, 2004).

Thyroid hormones are known to be involved in an increase in skeletal muscle protein expression, Na^+^/K^+^ ATPase activity, sarcoplasmic reticulum calcium ATPase expression, changes in fatty acyl composition of the cell membrane, and β-adrenergic receptor density (Hulbert, 2000; Harper and Seifert, 2008). Thyroid hormones also stimulate maturation of actin and myosin isoforms resulting in maturation of skeletal muscle contractile function in developing chicken and turkey (Gardahaut et al., 1992; Maruyama et al., 1995). Thyroid hormone mediated maturation of skeletal muscle enables increased capacity for shivering thermogenesis, which is present early in the development of endothermy in altricial Red-winged blackbirds (Olson, 1994). Thyroid hormones also act directly on the central nervous system, specifically the hypothalamus, modulating sympathetic outflow signaling to skeletal muscle and heart (McNabb, 2007). Maturation of these processes in the nestling are required to exhibit a strong endothermic response to cooling in the form of increased metabolic rate.

Hyperthyroid conditions early in the nestling stage did not influence metabolism or the development of endothermy from 5 dph onward. It was predicted that moving the rise in T_3_ to earlier in the nestling period would accelerate the development of endothermy, such that 5 dph hyperthyroid animals would exhibit at least a moderate endothermic response to cooling. However, the metabolism and endothermic response of 5 dph hyperthyroid nestlings was similar to control nestlings. While hyperthyroid conditions produced an increase in resting metabolic rate of adult birds (Liu et al., 2006; Ruuskanen et al., 2021), the influence on birds just after hatching seems to be limited. During hatching, precocial Pekin ducks exposed to elevated plasma T_3_ levels had elevated resting metabolisms compared with euthyroid embryos (Sirsat and Dzialowski, 2020). Red-winged blackbird nestlings experiencing hyperthyroid during the first 5 nestling days exhibited similar metabolic responses to cooling on 5, 7, and 9 dph as those that had been euthyroid throughout the first 5 days as nestlings (Figure 5). Contrary to our prediction, the findings suggest that elevated thyroid hormones prior to the natural elevation in plasma T_3_ are unable to accelerate the developmental trajectory. This observation may be because during this phase of development, tissue T_3_ levels are already high enough to fully saturate the thyroid hormone receptors.

On 9 dph, MMI-treated nestlings showed endothermic metabolic responses similar to control and T_3_-supplemented animals (Figure 3C, Figure 5C). These results confirm that hypothyroid status decreased resting ${\dot{V}o}_{2}$ and delayed the development of endothermic capacity in developing wild altricial red-winged blackbird. However, once the hypothyroid condition was removed, the nestlings could compensate, so that by 9 dph, treated birds had a similar ${\dot{V}o}_{2}$ response as the controls.

### Pulmonary ventilation

Development of ventilation responses to cooling in control nestlings develops along the same trajectory as that of metabolic rate. Control nestling red-winged blackbirds were able to increase minute ventilation in response to environmental cooling beginning on 7 dph. During cooling, 5 dph nestlings maintained tidal volume, but ventilation frequency decreased (Figures 3, 5). By day 9, nestlings increased minute ventilation with an increase in both tidal volume and breathing frequency in response to gradual cooling. Development of ventilation associated with an endothermic metabolic response has largely been examined in precocial birds (Sirsat and Dzialowski, 2016). In a similar fashion to the red-winged blackbird, the precocial pekin duck hatchling increased both tidal volume and breathing frequency to elevate minute ventilation during cooling (Sirsat and Dzialowski, 2016, Sirsat and Dzialowski, 2020). While an endothermic increase in ventilation does not occur until after 5 dph, chemosensitivity to oxygen and carbon dioxide develops well before this time in the red-winged blackbird (Dzialowski et al., 2016). Therefore, although nestlings at 5 dph have the capacity to finely regulate ventilation, they do not do so in response to cooling.

Altering the trajectory of the nestling plasma T_3_ levels had variable effects on the development of pulmonary ventilation and its regulation during gradual cooling. Elevating plasma T_3_ levels prior to the “normal” rise that occurs at 4-5 dph had no influence on the development of pulmonary ventilation. Ventilation control was altered when T_3_ levels were inhibited by administration of MMI during the first half of the nestling stage. Developing under hypothyroid conditions resulted in lower tidal volume, frequency, and ventilation at 5 dph and lower tidal volumes at rest in days 7 and day 9 nestlings (Figure 3). 9 dph hypothyroid animals were able to elevate ventilation frequency to offset the lower tidal volumes, resulting in similar ventilation as the control animals. On 7 dph, the MMI treated nestlings did not increase tidal volume like the control nestlings. This observation suggests a lack of T_3_ during this period of development may be influencing the development of the respiratory control network. It remains to be seen if hypothyroid conditions would also influence the development of the hypoxic and hypercapnic ventilatory responses (Dzialowski et al., 2016).

Precocial Pekin ducks exposed to hypothyroidism during the late stages of development exhibit similar changes in ventilation frequency, tidal volume, and minute ventilation (Sirsat and Dzialowski, 2020). Hypothyroidism in chicken embryos resulted in solid lung tissue on 19 days of incubation, a decrease in ventilation frequency, increase in thyroid receptor-β (TR), and attenuation of the natural increase in angiotensin converting enzyme (ACE) activity (Wittmann et al., 1987; Bjørnstad et al., 2016). Hypothyroidism in mammals resulted in decrease of central respiratory drive along with reduction in respiratory muscle strength (Milla and Zirbes, 2012). In the mammalian fetus, thyroid hormones play a role in pulmonary fluid resorption, increased expression of pulmonary β-adrenergic receptors, and increased Na^+^/K^+^ ATPase activity (Barker et al., 1990; Wilson et al., 2007; Das, 2013). In developing chicken, thyroid hormones affect lung blood flow, pulmonary vascular resistance through action on kallikrein-kinin, and ACE systems (Decuypere et al., 2005).

Delays in pulmonary ventilation caused by hypothyroidism have been observed in developing rats. Rat pups whose mothers were treated with MMI from initiation of pregnancy through 2 weeks post postpartum exhibited altered ventilation function (Rousseau et al., 2019). In both neonatal birds and mammals, hypothyroidism has a negative influence on ventilation development that may be due to alterations of the autonomic nervous system during development. Hypothyroid conditions delayed the maturation of the respiratory control network in rat pups (Rousseau et al., 2021). In the rat pups, there was a GABAergic inhibition that produced the abnormal respiratory responses. These same GABAergic pathways may play a role in the avian response observed here. Hypothyroid conditions in neonatal rats resulted in degradation and hypertrophy of the diaphragm muscles (Canavan et al., 1994). The mechanism by which hypothyroidism influences development of respiratory control of altricial birds remains to be seen.

## Conclusion

In the altricial red-winged blackbird nestling, the trajectory of thyroid hormone levels during early development influences growth and metabolic maturation. In this study, greater developmental changes were seen in response to hypothyroidism than hyperthyroidism. Decreased levels of plasma T_3_ in the current study showed, for the first time in wild altricial birds, delayed ontogeny of endothermic response. These findings are similar to delayed endothermic development observed in developing precocial Pekin duck in response to hypothyroidism (Sirsat and Dzialowski, 2020). The lack of metabolic and ventilatory changes in response to hyperthyroidism may be due to the already high levels of T_3_ during development, with further elevation having little effect as receptors may already be saturated.

## Data Availability

The raw data supporting the conclusion of this article will be made available by the authors, without undue reservation.
